# Biomedical named entity extraction: some issues of corpus compatibilities

**DOI:** 10.1186/2193-1801-2-601

**Published:** 2013-11-12

**Authors:** Asif Ekbal, Sriparna Saha, Utpal Kumar Sikdar

**Affiliations:** Department of Computer Science and Engineering, Indian Institute of Technology, Patna, India

## Abstract

**Background:**

Named Entity (NE) extraction is one of the most fundamental and important tasks in biomedical information extraction. It involves identification of certain entities from text and their classification into some predefined categories. In the biomedical community, there is yet no general consensus regarding named entity (NE) annotation; thus, it is very difficult to compare the existing systems due to corpus incompatibilities. Due to this problem we can not also exploit the advantages of using different corpora together. In our present work we address the issues of corpus compatibilities, and use a single objective optimization (SOO) based classifier ensemble technique that uses the search capability of genetic algorithm (GA) for NE extraction in biomedicine. We hypothesize that the reliability of predictions of each classifier differs among the various output classes. We use Conditional Random Field (CRF) and Support Vector Machine (SVM) frameworks to build a number of models depending upon the various representations of the set of features and/or feature templates. It is to be noted that we tried to extract the features without using any deep domain knowledge and/or resources.

**Results:**

In order to assess the challenges of corpus compatibilities, we experiment with the different benchmark datasets and their various combinations. Comparison results with the existing approaches prove the efficacy of the used technique. GA based ensemble achieves around 2% performance improvements over the individual classifiers. Degradation in performance on the integrated corpus clearly shows the difficulties of the task.

**Conclusions:**

In summary, our used ensemble based approach attains the state-of-the-art performance levels for entity extraction in three different kinds of biomedical datasets. The possible reasons behind the better performance in our used approach are the (i). use of variety and rich features as described in Subsection “Features for named entity extraction”; (ii) use of GA based classifier ensemble technique to combine the outputs of multiple classifiers.

## Background

Named Entity (NE) extraction is one of the most fundamental and important tasks in biomedical information extraction. This involves two different stages, i.e. identification of certain kinds of entities and classification of them into some predefined categories. This overall task is termed as Named Entity Recognition and Classification (NERC). Biomedical named entities (NEs) include mentions of proteins, genes, DNA, RNA etc. which, in general, have complex structures and so difficult to recognize. The supervised approaches (Finkel et al. 2004; GuoDong and Jian 2004; Kim et al. 2005; Settles 2004; Wang et al. 2008) have been widely used for NERC in biomedical texts. The release of tagged GENIA corpus (Ohta et al. 2002) provides a way of comparing the existing biomedical NERC systems. However, most of these state-of-the-art approaches suggest that individual system may not cover entity representations with arbitrary set of features and cannot achieve best performance. There exists other two benchmark datasets, namely AIMED^1^ and GENETAG^2^.

The existing corpora do not have an uniform rule of annotation, and so they are not compatible to each other. Thus it is not possible to use all the available corpora together for building any supervised NE extraction system. This reduces to two different problems, *viz.* (i). it is hard to compare systems which are created using different corpora and (ii). there is hardly any existing state-of-the-art NE extraction system which can perform equally well for many domains.

Classifier ensemble (Ekbal and Saha 2010a; 2010b; 2011a; 2012) is an important research topic in machine learning in recent years. It is an effective method to increase the generalization accuracy by combining the outputs of different classifiers. In the present work, we use a single objective optimization (SOO) based classifier ensemble technique proposed in (Ekbal and Saha 2011b). In SOO, we optimize a single classification quality measure (i.e. objective function) such as recall, precision or F-measure at a time. Here, we optimize F-measure which is the harmonic mean of recall and precision both. This optimization technique is based on genetic algorithm (GA) (Goldberg 1989) which is a randomized search and optimization technique guided by the principles of evolution and genetics, having a large amount of implicit parallelism.

In the present work we assess the challenges of corpus incompatibilities using three existing benchmark datasets, namely JNLPBA 2004 shared task (Jin-Dong et al. 2004), AIMed^3^ and GENETAG^4^, (Saha and Ekbal 2011). At first we experiment with the original datasets. Thereafter, we consider different experimental settings by considering the various combinations of these datasets. This work is inspired from the work of cross corpus utilization reported in (Wang et al. 2009). However, it is to be noted that our approach significantly differ from (Wang et al. 2009) in terms of proposed technique and experimental settings. Conditional Random Field (CRF) and Support Vector Machine (SVM) are used as the base classifiers. Various models of these two classifiers are constructed by varying the available features and/or feature templates. We identify a very rich and effective feature set that includes variety of features based on orthography, local contextual information and global contexts. One most important characteristic of our system is that the *identification and selection of features are mostly done without using any deep domain knowledge and/or resources*. Our main focus is to investigate the appropriate weights for voting rather than searching for the best performing individual models. Degradation in performance on the integrated corpus clearly indicates the challenges in building an ideal system that could perform almost at the same levels across many domains.

The present work also differs from the previous works reported in (Ekbal and Saha 2010a; 2010b; 2011a; 2011b; 2012; Saha and Ekbal 2011). In (Ekbal and Saha 2010a), a GA based classifier ensemble selection technique was developed. This approach determines only a subset of classifiers that can form the final classifier ensemble, and the proper weights of votes for all the classes were not determined. In (Ekbal and Saha 2010b; 2011b) a GA based technique was developed for weighted vote based classifier ensemble selection. The extended version of this work is reported in (Ekbal and Saha 2011b), where the present methodology is more elaborately discussed, evaluated on multiple languages and compared against the existing popular methods. In addition a GA based feature selection technique was also introduced. In (Ekbal and Saha 2012) a multiobjective optimization based technique is developed for classifier ensemble. Along with feature selection technique exhaustive evaluation was carried out. In (Ekbal and Saha 2011a) a multiobjective (MOO) technique is developed for weighted voted classifier ensemble selection. Here the search capability of a newly developed simulated annealing based MOO technique, AMOSA (Bandyopadhyay et al. 2008) is used as the underlying optimization technique. Several different versions of the objective functions are exploited. In (Saha and Ekbal 2011), a SVM based gene mention detection technique is developed. Based on the different feature representations many classifiers were generated. At the end these were combined using simple majority and weighted voting approaches. The technique was evaluated only for the GENTAG data set.

We highlight the differences from the previous works reported in (Ekbal and Saha 2010a; 2010b; 2011a; 2011b; 2012; Saha and Ekbal 2011) as below:

1. The work reported in this paper deals with the problems of information extraction, especially NE extraction in biomedical domain, which is more difficult and challenging. The inherent structures of the biomedical entities pose a big challenge for their identification. Moreover, they hardly follow any standard nomenclature.
2. Compared to (Saha and Ekbal 2011), many new features are introduced and implemented. In this paper our main aim was to come up with a system that could perform satisfactorily on different kinds of datasets. Compared to the previously published work we present more systematic evaluations on different combinations of the datasets.
3. The present work discusses the crucial issue of corpus incompatibilities. It is often the fact that any system developed targeting any domain or language does not perform well for other domains or languages. Therefore, there is a great demand of designing some systems that could achieve good accuracies on many corpora that were annotated following different guidelines.
4. As an evidence that our present approach is not biased to any particular domain, firstly we present detailed evaluation results on three benchmark datasets separately; and secondly we evaluate on the different combinations of these datasets. Our current approach attains encouraging performance in all the settings.

## Results and discussion

In this section, we present the descriptions of performance metrics, datasets, experimental setup, and report the detailed evaluation results of our approach.

### Performance measures

All the classifiers are evaluated in terms of recall, precision and F-measure. We use the same strict matching criterion that was followed in the JNLPBA 2004 shared task evaluation and used their script^5^. The full credit is given if and only if both the left and right boundaries match. Precision is the ratio of the number of correctly found *NE chunks* (i.e., more than one token) to the number of found NE chunks, and recall is the ratio of the number of correctly found NE chunks to the number of true NE chunks.

### Datasets and experimental setup

We evaluate our approach with three benchmark datasets, namely JNLPBA 2004 shared task^6^, AIMed and GENETAG. The JNLPBA datasets were extracted from the GENIA Version 3.02 corpus of the GENIA project. This was constructed by a controlled search on Medline using MeSH terms such as *human*, *blood cells* and *transcription factors*. From this search, 2000 abstracts of about 500K wordforms were selected and manually annotated according to a small taxonomy of 48 classes based on a chemical classification. Out of these classes, 36 classes were used to annotate the GENIA corpus. In the shared task, the data sets were further simplified to be annotated with only five NE classes, namely *Protein*, *DNA*, *RNA*, *Cell_line* and *Cell_type* (Jin-Dong et al. 2004). The test set was relatively new collection of Medline abstracts from the GENIA project. The test set contains 404 abstracts of around 100K words. One half of the test data was from the same domain as that of the training data and the rest half was from the super domain of *blood cells* and *transcription factors*. For simplification, embedded structures were removed leaving only the outermost structures (i.e. the longest tag sequence). Consequently, a group of coordinated entities involving ellipsis were annotated as one structure like in the following example: ⋯ in [ *lymphocytes*] and [ *T*- and B - lymphocyte ] count in ⋯

In the example, 'T- and B-lymphocyte’ was annotated as one structure but involves two entity names, 'T-lymphocyte’ and 'B-lymphocyte’, whereas 'lymphocytes’ was annotated as one and involves as many entity names. In order to properly denote the boundaries of NEs, five classes are further divided using the BIO format, where 'B-XXX’ refers to the beginning of a multi-word/single-word NE of type 'XXX’, 'I-XXX’ refers to the rest of the words of the NE and 'O’ refers to the entities outside the NE. For each of these B- and I- type classes we calculate the appropriate weight using GA and construct the ensemble.

Like GENIA^7^, AIMed also focuses on the human domain, and exhaustively collect sentences from the abstracts of PubMed. But, it selects the different text spans for protein annotation. In GENIA, almost always the word '*protein*’ is included as part of protein annotation. But, in most cases, this word is not included as part of the protein name in AIMed. This ambiguous annotation of boundary is a crucial factor and affects the average length of protein mentions, and this could be a major source of performance degradation when combined with other corpus.

The protein annotations in GENIA corpus is based on the definitions of GENIA ontology (Ohta et al. 2002). In GENIA, other than protein classes (for e.g., DNA, RNA etc.), the subclasses of protein are also included. Unlike GENIA, protein families are not annotated in AIMed. In AIMed, tagging is done for only those specific names which could ultimately be traced back to specific genes in the human genome (Buescu et al. 2005). For example,“tumor necrosis factor” was not tagged while “tumor necrosis factor alpha” was annotated. Some gene names without differentiating them from proteins are included in the annotations in AIMed. In GENIA, 'protein’ tags were associated only to proteins, while genes were associated in the scope of DNA annotations. The AIMed corpus consists of 225 abstracts that contain 1,987 sentences with 4,075 protein mentions. Here 1.3 tokens are there in the protein mentions in an average. In order to properly denote the boundaries of proteins, we use the same BIO notations that were followed in GENIA. Unlike GENIA and AIMed, GENETAG covers a more general domain of PubMed. It contains both true and false gene or protein names in a variety of contexts. In GENETAG, not all the sentences of abstracts were included, rather more NE informative sentences were considered. In terms of text selection, GENIA and GENETAG are closer to each other, compared to AIMed. GENIA and GENETAG selected longer text fragments as entity reference. Like GENIA, GENETAG also includes the semantic category word 'protein’ for protein annotation.

We evaluate our approach with the GENETAG training and test datasets, available at the site^8^. Gene mentions in both the training and test datasets were annotated with the 'NEWGENE’ tag and the overlapping gene mentions were distinguished by another tag 'NEWGENE1’. However, in this work, we use the standard BIO notations (as in GENIA corpus) to properly denote the boundaries of gene names, and we replace all the 'NEWGENE1’ tags by 'NEWGENE’ for training and testing. The training dataset contains 7,500 sentences with 8,881 gene mentions. The average length per protein mention is 2.1 tokens. The test dataset consists of 2,500 sentences with 2,986 gene mentions. The system is evaluated using the evaluation script that was provided by the BioCreative-II^9^ evaluation challenge for the gene mention detection task.

The individual models based on CRF and SVM are generated using CRF ^++^ package^10^ and YamCha^11^ toolkit, respectively. For CRF training, we use CRF ^++^ 0.54 version and set the following parameter values, regularization parameter (a): default setting, i.e. L2; soft-margin parameter (c): trades the balance between overfitting and underfitting (default value); and cut-off threshold for the features (f): uses the features that occurs no less than its value in the given training data (set to 1, i.e. all the features that appear at least once in the training dataset is considered). We develop our system using SVM (Joachims 1999; Vapnik 1995) which performs classification by constructing an N-dimensional hyperplane that optimally separates data into two categories. We have used YamCha^12^ toolkit, an SVM based tool for detecting classes in documents and formulating the NE extraction task as a sequential labeling problem. Here, the *pairwise multi-class decision* method and the *polynomial kernel function* are used. We use TinySVM-0.07^13^ classifier for classification. We set the following parameter values for GA: population size=100, number of generations=50, probability of mutation and crossover are determined adaptively.

### Results on the original corpus

In this section, we report the evaluation results with the original datasets of GENIA, GENTAG and AIMed (Saha et al. 2013). We build many CRF and SVM based classifiers by varying the various available features.

We report the evaluation results of the approach along with the best individual classifiers in Table 1. For GENIA corpus the best individual classifier produces the best recall, precision and F-measure values of 73.10%, 76.78% and 74.90%, respectively. This corresponds to a CRF based classifier with the following feature template: the contexts of previous and next two tokens and their all possible n-gram (*n*≤2) combinations from left to right, prefixes and suffixes of length up to 3 characters of only the current word, feature vector consisting of length, infrequent word, normalization, chunk, orthographic constructs, Part-of-Speech (PoS), trigger word, semantic information, unknown word, head noun, word class, effective NE information of only the current token, and bigram feature combinations. For AIMed corpus the best individual model corresponds to a SVM classifier and it shows the average recall, precision and F-measure values of 94.56%, 92.66% and 93.60%, respectively. The feature combinations are the contexts of previous and next three tokens and their all possible n-gram (*n* ≤ 2) combinations from left to right, prefixes and suffixes of length up to 4 characters of only the current word, feature vector consisting of length, infrequent word, normalization, chunk, orthographic constructs, trigger word, semantic information, unknown word, head noun, word class, effective NE information of only the current token, and dynamic NE information of previous three tokens. For GENETAG, the highest performance corresponds to a CRF based classifier that yields the overall recall, precision and F-measure values of 95.35%, 95.31% and 95.33%, respectively. The following feature template is used to generate it: contexts of previous and next one token and their all possible n-gram (*n* ≤ 1) combinations from left to right, prefixes and suffixes of length upto 4 characters of only the current word, feature vector consisting of length, infrequent word, normalization, chunk, orthographic constructs, PoS of current word, previous two words and next two words, unknown word, head noun, word class, effective NE information of only the current token, and bigram feature combinations. Please note that for AIMed and GENETAG datasets we used our in-house NE extractor for getting the class label information of the test data for computing the “content words in surrounding contexts” feature.

**Table 1:** **Overall evaluation results (we report percentages) on the original corpus (**Saha et al. 2013 **)**

Corpus	Model	Recall	Precision	F-measure
GENIA	Best individual classifier	73.10	76.78	74.90
	SOO based ensemble	74.17	77.87	75.97
AIMeD	Best individual classifier	94.56	92.66	93.60
	SOO based ensemble	95.65	94.23	94.93
GENETAG	Best individual classifier	95.35	95.31	95.33
	SOO based ensemble	95.99	95.81	95.90

Finally the SOO based ensemble selection technique (Ekbal and Saha 2011b) is used to combine the outputs of all these individual classifiers. Results of these SOO based approaches are also shown in Table 1. We achieve the Increments of 1.07%, 1.33% and 0.57% over the individual classifiers for GENIA, AIMed and GENETAG, respectively.

We compare the performance of our developed system with some other biomedical entity extraction systems that made use of the same datasets, i.e. GENTAG. We compare with the systems reported in the BioCreative-2 challenges as well as with those that were developed at the later stages but made use of the same datasets. Almost all the features were automatically extracted from the training dataset. In our experiment, we use only PoS, chunk (or, phrase) and an external NE extractor as the domain dependent knowledge sources. We present the comparative evaluation results in Table 2 not only with the domain-independent systems but also with the systems that incorporate deep domain knowledge and/or external resources. Our current approach attains an improvement of more than 14% over the existing state-of-the-art approaches. We systematically analyze the contribution of each feature, and it reveals the fact that huge performance gain is achieved with the PoS information which was provided with the dataset. After observing this remarkable performance gain we analyzed each step of our implementation thoroughly. It seems that one possible explanation behind this radical improvement could be as follows. It is to be noted that in the GENETAG training and test datasets, PoS information were provided only for the non-gene proteins. We preprocessed this data and assigned the PoS class, NNP, i.e. proper noun to each of these gene tokens. This PoS information actually plays a crucial role in the overall system performance. Another reason is that we used our in-house NE extractor for getting the class label information of the test data for computing the “content words in surrounding contexts” feature.

**Table 2:** **Comparison with the existing approaches for GENETAG data set**

System	Approach used	Domain knowledge/resources	F-measure
Our system	GA based ensemble	PoS, phrase	94.70
	(CRF and SVM)		
Song et al. (2005) (Song et al. 2004a)	SVM	-	66.7
Bickel et al. (2004) (Bickel et al. 2004)	SVM	a dictionary	72.1
Kinoshita et al. (2005) (Kinoshita et al. 2005)	TnT (Brants 2000), the Trigrams ’n’ Tags	dictionary based postprocessing	80.9
		HMM-based part-of-speech tagger	
Mitsumoriet al. (2005) (Mitsumori et al. 2005)	SVM	gene/protein name dictionary	78.09
Finkel et al. (2004) (Finkel et al. 2005)	ME+ post processing		82.2
McDonald and Pereira (2005) (McDonald and Pereira 2005)	CRF		82.4
GuoDong et al. (2005) (Zhou and Su 2002)	HMM, SVM, Ensemble technique	Post processing	82.58

Next, we compare the performance of our current system with other biomedical entity extraction systems that made use of the same GENIA dataset. We compare with the systems, developed with same datasets. Our system does not make use of any deep domain knowledge and/or external resources. In our experiment, we use only PoS and chunk (or, phrase) information as the domain dependent knowledge. So, it will not be fair to compare the performance of ensemble based system with all the available systems. However, we present the comparative evaluation results in Table 3 not only with the domain-independent systems but also with the systems that incorporate deep domain knowledge and/or external resources.

**Table 3:** **Comparison with the existing approaches for GENIA data set**

System	Used approach	Domain knowledge/resources	FM
Our system	Classifier ensemble	POS, phrase	76.52
	(CRF and SVM)		
Zhou & Su (2004) (GuoDong and Jian 2004) Final	HMM, SVM	Name alias, cascaded NEs dictionary, POS, phrase	72.55
Zhou & Su (2004) (GuoDong and Jian 2004)	HMM, SVM	POS, phrase	64.1
Kim et al. (2005) (Kim et al. 2005)	Two-phase model	POS, phrase,	71.19
	with ME and CRF	rule-based component	
Finkel et. al (2004) (Finkel et al. 2004)	CRF	Gazetteers, web-querying, surrounding abstracts,	70.06
		abbreviation handling, BNC corpus, POS	
Settles (2004) (Settles 2004)	ME	POS, semantic knowledge sources of 17 lexicons	70.00
Saha et al. (2009) (Saha et al. 2009)	ME	POS, phrase	67.41
Park et. al (2004) (Park et al. 2004)	ME	POS, phrase, domain-salient words using WSJ,	66.91
		morphological patterns, collocations from Medline	
Song et al. (2004) (Song et al. 2004b) Final	SVM, CRF	POS, phrase, Virtual sample	66.28
Song et al. (2004) (Song et al. 2004b) Base	SVM	POS, phrase	63.85
Ponomareva et al. (2007) (Ponomareva et al. 2007)	HMM	POS	65.7

Zhou and Su (GuoDong and Jian 2004) developed the best system in the JNLPBA 2004 shared task. This system provides the highest F-measure value of 72.55 with several deep domain knowledge sources. But when the system used only PoS and chunk information as the domain knowledge, the F-measure value drops to 64.1%. Song et al. (Song et al. 2004b) used CRF and SVM both, and obtained the F-measure of 66.28% with virtual samples. The HMM-based system reported by Ponomareva et al. (Ponomareva et al. 2007) achieved a F-measure value of 65.7% with PoS and phrase-level domain dependent knowledge. A ME-based system was reported in (Park et al. 2004) where recognition of terms and their classification were performed in two steps. They achieved a F-measure value of 66.91% with several lexical knowledge sources such as salient words obtained through corpus comparison between domain-specific and WSJ corpora, morphological patterns and collocations extracted from the Medline corpus. As far our knowledge is concerned, one of the very recent works proposed in (Saha et al. 2009) obtained the F-measure value of 67.41% with PoS and phrase information as the only domain knowledge. This is the highest performance achieved by any system that did not use any deep domain knowledge.

A CRF-based NE extraction system has been reported in (Settles 2004) that obtained the F-measure value of 70% with orthographic features, semantic knowledge in the form of 17 lexicons generated from the public databases and Google sets. Finkel et al. (Finkel et al. 2004) reported a CRF-based system that showed the F-measure value of 70.06% with the use of a number of external resources, including gazetteers, web-querying, surrounding abstracts, abbreviation handling method, and frequency counts from the BNC corpus. A two-phase model based on ME and CRF was proposed by Kim et al. (Kim et al. 2005) that achieved a F-measure value of 71.19% by postprocessing the outputs of machine learning models with a rule-based component. We also compare the performance of our developed ensemble based approach with BANNER (Leaman and Gonzalez 2008) that was implemented using CRFs. BANNER exploits a range of orthographic, morphological and shallow syntax features, such as part-of-speech tags, capitalisation, letter/digit combinations, prefixes, suffixes and Greek letters. Comparisons between the several existing NE extraction systems are provided in (Kabiljo et al. 2009). For BANNER, Kabiljo et al. (Kabiljo et al. 2009) reported the F-measure values of 77.50% and 61.00% under the sloppy matching and strict matching criterion, respectively with the JNLPBA shared task datasets.

In summary, our developed ensemble based approach (Ekbal and Saha 2011b) attains the state-of-the-art performance levels for entity extraction in three different kinds of biomedical datasets. The possible reasons are the effient use of a diverse set of featuers and the utilization of the GA based ensmeble technique (Ekbal and Saha 2011b).

### Results on cross corpus

In this section we investigate the effects of corpus incompatibility on the NE extraction problem. In order to check whether our system performs reasonably well across various domains, we perform a series of experiments with the various combinations of the available corpora. Depending upon the nature of the datasets, we replace the corresponding annotations in the GENIA (i.e., JNLPBA) corpus. We describe below the different experimental setups.

1. Experiment-1: In the first experiment, we replace all other tags except 'Protein’ by 'O’ (other-than-NE) tags in the GENIA corpus, and added to the AIMed corpus. Three-fold cross validation experiments are carried out to report the evaluation results.
2. Experiment-II: In the second experiment, we keep only 'Protein’ and 'DNA’ annotations in GENIA corpus, and replace all the other annotations by 'O’. This corpus is integrated with the AIMed corpus, and 3-fold cross validation experiments are done to report the evaluation results.
3. Experiment-III: In the third experiment, all other annotations except 'Protein’ are replaced by 'O’ tags in the GENIA corpus. This is integrated with the GENETAG training corpus. Evaluation results are reported on the GENETAG test corpus.
4. Experiment-IV: In the fourth experiment, we keep only the 'Protein’, 'DNA’ and 'RNA’ annotations in the GENIA corpus. This corpus is integrated with the GENETAG corpus, and this resultant corpus is used for training. Evaluation results are reported on the original GENETAG test corpus.

We generate several different versions of CRF and SVM based classifiers by considering various subsets of the available features. Here we report only the performance of the best individual classifier. Results of all these experiments are reported in Table 4. It shows the best performance with a CRF classifier in all the four experiments.

**Table 4:** **Evaluation results of the approach on cross-corpus datasets (we report percentages); Here 'FM’ denotes 'F-measure’**

Approach	Training set	Test set	Recall	Precision	FM
Best Ind. Classifier	JNLPBA (protein only)+AIMed	AIMed	83.14	83.19	83.17
SOO	JNLPBA (protein only)+AIMed	AIMed	85.10	85.01	85.05
Best Ind. Classi	JNLPBA (protein + DNA)+AIMed	AIMed	82.17	84.15	83.15
SOO	JNLPBA (protein + DNA)+AIMed	3-fold cross	84.07	86.01	85.03
		validation on AIMed			
Best Ind. Classi	JNLPBA (protein only)+GENETAG	GENETAG	89.44	93.07	91.22
SOO	JNLPBA (protein only)+GENETAG	GENETAG	91.19	94.98	93.05
Best Ind. Classi	JNLPBA (protein + DNA + RNA)+GENTAG	GENTAG	88.70	93.55	91.06
SOO	JNLPBA (protein + DNA + RNA)+GENTAG	GENTAG	90.09	95.16	92.56

Finally we apply our single objective GA based ensemble technique (Ekbal and Saha 2011b) to combine the results of all the individual base classifiers. In all our experimental settings, we observe that our approach performs superior compared to all the individual classifiers. It attains the performance improvements of 1.88, 1.37, 1.50 and 1.83 F-measure points over the four best individual classifiers, respectively. Comparison between Table 1 and Table 4 clearly show that due to corpus incompatibility performance drops significantly when GENIA is added to AIMed. When only the protein annotation is retained and others are replaced by non-NE tags, the overall performance drops by 9.88 percentage F-measure points (c.f. results of Experiment-I). This, in turn, decreases the overall performance. Overall performance further drops when we consider DNA annotations, in addition to 'Protein’ (c.f. results of Experiment-II). But, in each of these two cases, the system performs superior in comparison to the performance reported in (Wang et al. 2009). Similarly, we also observe the drops in accuracies (comparing between Table 1 and Table 4) in case of GENIA and GENETAG. However, it is to be noted that the performance drops are very minor in comparison to AIMed. Overall F-measure values decrease by only 1.85 and 3.34 percentage points in the third and fourth experiments, respectively. Thus, we can conclude that these corpora, i.e. GENIA and GENETAG are more compatible to each other.

Our datasets are imbalanced. In order to make the ratio of positive and negative examples more compatible, we remove the sentences that don’t contain any gene/protein names from the combined corpora. Evaluation results of these sampled corpora are reported in Table 5. Comparisons between the results of Table 4 and Table 5 show that, in general, the performance improves due to the removal of non-informative sentences from the AIMed, GENETAG and GENIA corpora. However for the first experiment (i.e., GENIA + AIMed), we observe a little drop (1.10 percentage F-measure points) in the overall performance. But it is to be noted that when DNA annotation is also considered along with 'Protein’ annotation, performance increases by 0.51 percentage F-measure points (c.f. results of Experiment-II). Thus, we can conclude that removal of non-informative sentences from the training corpora sometimes helps to improve system performance.

**Table 5:** **Evaluation results of the approach on cross-corpus non-informative sentence-removed datasets (we report percentages)**

Approach	Training set	Test set	r	p	FM
Best Individual Classifier	JNLPBA (protein only)+AIMed	AIMed	80.58	84.43	82.46
SOO Based Ensemble	JNLPBA (protein only)+AIMed	AIMed	81.98	86.01	83.95
Best Individual Classifier	JNLPBA (protein + DNA)+AIMed	AIMed	84.66	83.50	84.08
SOO Based Ensemble	JNLPBA (protein + DNA)+AIMed	AIMed	86.07	85.01	85.54
Best Individual Classifier	JNLPBA (protein only)+GENETAG	GENETAG	91.79	90.61	91.20
SOO Based Ensemble	JNLPBA (protein only)+GENETAG	GENETAG	93.19	92.08	92.63
Best Individual Classifier	JNLPBA (protein + DNA + RNA)+GENTAG	GENTAG	93.98	90.67	92.29
SOO Based Ensemble	JNLPBA (protein + DNA + RNA)+GENTAG	GENTAG	95.09	92.16	93.60

Here 'r’: recall, 'p’: precision, 'FM’: F-measure.

We also compare our approach with the results obtained by (Wang et al. 2009). They attained the recall, precision and F-measure values of 65.06%, 67.31% and 66.16%, respectively for the experiment similar to our first experiment. Similarly for the other three experiments they reported the overall F-measure values of 55.76%, 63.62% and 48.21%, respectively. Thus, for all kinds of experiments our approach attains better performance.

## Conclusions

In this paper we have assessed the challenges associated in using more than one corpus for biomedical named entity extraction. The challenges are mainly due to the different annotation schemes followed by the different groups. One of the major motivation of this work was to come up with a system that could achieve good accuracies for many domains. We identified and developed a very rich feature set that mostly contains the domain-independent features. Due to this domain-independent nature we were able to apply these features on many benchmark datasets. Initially we have generated individual classifiers varying these feature combinations. We have used CRF and SVM frameworks as the base classifiers. Later on outputs of these classifiers are combined using a single objective classifier ensemble selection technique. This classifier ensemble technique is based on genetic algorithm (Ekbal and Saha 2011b), a randomized search and optimization technique guided by natural evolution and genetics. Experiments with the benchmark datasets like JNLPBA, AIMed and GENETAG show that our developed approach attains state-of-the-art accuracies. We also performed a series of experiments by considering various combinations of the benchmark datasets. Our preliminary experiments revealed the complexities associated with the compatibilities of protein annotations across the corpora with the performance degradations by significant margins (according to the exact matching criterion, the *F-measure* values decreased by about 9.88% on AIMed and 3.34% on GENETAG). Experiments with the removal of non-informative sentences (i.e. sentences that don’t contain any protein or gene names) from the training showed some performance improvements. Results also showed that our system achieves the state-of-the-art accuracies for all these cases.

## Methods

In this section we first formulate the weighted vote based classifier ensemble selection problem and thereafter discuss about the genetic algorithm (GA) based classifier ensemble technique (Ekbal and Saha 2011b) to solve this particular problem.

### Weighted vote based classifier ensemble problem formulation

The weighted vote based classifier ensemble problem (Ekbal and Saha 2010b) is stated below. Suppose, the *N* number of available classifiers be denoted by *C*_1_,…,*C*_*N*_. Let, . Suppose, there are *M* output classes. The weighted vote based classifier ensemble problem is then stated as follows:

Find the weights of votes *V* per classifier which will optimize some function *F*(*V*). Here, *V* is an real array of size *N*×*M*. *V*(*i*,*j*) denotes the weight of vote of the *i*^*th*^ classifier for the *j*^*th*^class. More weight is assigned for that particular class for which the classifier is more confident; whereas the output class for which the classifier is less confident is given less weight. These weights are used while combining the outputs of classifiers using weighted voting. Here, *F*_*i*_s are some classification quality measures of the combined weighted vote based classifier. The particular type of problem like NE extraction has mainly three different kinds of classification quality measures, namely recall, precision and F-measure. Thus, *F*∈{recall,precision,F-measure}.

The weighted vote based classifier ensemble problem can be formulated under the single objective optimization (SOO) framework as below: For each classifier, find the weights of votes *V* per classifier such that, *maximize* [ *F*(*V*)], where *F*∈{recall,precision,F-measure}. We choose *F*=F-measure, which is the harmonic mean of recall and precision both.

### Methodology

Below we describe the genetic algorithm based classifier ensemble technique (Ekbal and Saha 2011b) used in the current works. This technique is applied to combine the outputs of multiple classifiers. The steps of the genetic algorithm based classifier ensemble technique are as follows.

#### String representation and population initialization

This string representation scheme is very similar to that developed in (Ekbal and Saha 2010b;, 2011b). Suppose, there are *N* available classifiers and *O* output classes. Then, the length of the chromosome is *N*×*O*. Each chromosome encodes the weights of votes for possible *O* output classes^14^for each classifier. We use real encoding that randomly initializes the entries of each chromosome by a real value (r) between 0 and 1. Here, *r* is an uniformly distributed random number between 0 and 1. If the population size is *P* then all the *P* number of chromosomes of this population are initialized in the above way.

#### Fitness computation

Initially, the F-measure values of all the classifiers are calculated using 5-fold cross validation. Each of these classifiers is built using various representations of the available features and/or feature templates. Thereafter, we execute the following steps to compute the fitness value of each chromosome.

1. Let, the overall F-measure values of the *N* number of classifiers be *F*_*i*_, *i*=1…*N*.
2. Initially, the training data is equally divided into 5 parts. Each classifier is trained using 4/5 portions of the training data and evaluated with the remaining 1/5 part. Now, for the ensemble classifier the output class for each token in the 1/5 training data is determined using the weighted voting of these *N* classifiers’ outputs. The weight of the output class provided by the *m*^*th*^ classifier is equal to *I*(*m*,*i*)×*F*_*m*_. Here, *I*(*m*,*i*) is the entry of the chromosome corresponding to *m*^*th*^classifier and *i*^*th*^output class. The combined score of a particular class *c*_*i*_for a particular token *t* is:
  Here, *op*(*t*,*m*) denotes the output class provided by the *m*^*th*^classifier for the token *t*.
  The class receiving the maximum combined score is selected as the joint decision. Note that in case different boundaries are outputted by the distinct classifiers, the final output is decided by the maximum combined score.
3. The overall F-measure value of the ensemble for the 1/5 part is calculated.
4. Steps 2 and 3 are repeated 5 times to perform 5-fold cross validation.
5. The average F-measure value of this 5-fold cross validation is used as the fitness value of the particular chromosome. This fitness function, *fit*=F-measure_*avg*_is maximized using the search capability of GA.

### Genetic operators

Roulette wheel selection is used to implement the proportional selection strategy. We use the normal single point crossover (Holland 1975). Crossover probability is selected adaptively as in (Srinivas and Patnaik 1994). The expressions for crossover probabilities are computed as follows:

Let *f*_*max*_be the maximum fitness value of the current population,  be the average fitness value of the population and  be the larger of the fitness values of the solutions to be crossed. Then the probability of crossover, *μ*_*c*_, is calculated as:

Here, as in (Srinivas and Patnaik 1994), the values of *k*_1_and *k*_3_are kept equal to 1.0.

Each chromosome undergoes mutation with a probability *μ*_*m*_. The mutation probability is also selected adaptively for each chromosome as in (Srinivas and Patnaik 1994). The expression for mutation probability, *μ*_*m*_, is given below:

Here, values of *k*_2_and *k*_4_are kept equal to 0.5. This adaptive mutation helps GA to come out of local optimum.

Here, each position in a chromosome is mutated with probability *μ*_*m*_in the following way. The value is replaced with a random variable drawn from a Laplacian distribution, , where the scaling factor *δ* sets the magnitude of perturbation. Here, *μ* is the value at the position which is to be perturbed. The scaling factor *δ* is chosen equal to 0.1. The old value at the position is replaced with the newly generated value. By generating a random variable using Laplacian distribution, there is a non-zero probability of generating any valid position from any other valid position while probability of generating a value near the old value is more.

### Termination condition

In this approach, the processes of fitness computation, selection, crossover, and mutation are executed for a maximum number of generations. The best string seen up to the last generation provides the solution to the above classifier ensemble problem. Elitism is implemented at each generation by preserving the best string seen up to that generation in a location outside the population. Thus on termination, this location contains the best classifier ensemble.

### Features for named entity extraction

Feature selection plays an important role for the success of machine learning techniques. We use a large number of following features for constructing the various models based on CRF and SVM classifiers (Saha and Ekbal 2011).

These features are general in nature and can be applied for other domains as well as languages. Due to the use of variety of features, the individual classifiers achieve very high accuracies.

1. **Context words**: These are the words occurring within the context window ,  and , where *w*_*i*_is the current word.
2. **Word prefix and suffix**. These are the word prefix and suffix character sequences of length up to *n*. The sequences are stripped from the leftmost (prefix) and rightmost (suffix) positions of the words.
3. **Word length**. We define a binary valued feature that fires if the length of *w*_*i*_is greater than a pre-defined threshold. Here, the threshold value is set to 5. This feature captures the fact that short words are likely not to be NEs.
4. **Infrequent word**. A list is compiled from the training data by considering the words that appear less frequently than a predetermined threshold. The threshold value depends on the size of the dataset. Here, we consider the words having less than 10 occurrences in the training data.
5. **Part of Speech (PoS) information**: PoS information is a critical feature for NE identification. In this work, we use PoS information of the current and/or the surrounding token(s) as the features. This information is obtained using GENIA tagger^15^V2.0.2, which is used to extract PoS information from the biomedical domain.
6. **Chunk information**: We use GENIA tagger V2.0.2 to get the chunk information. Chunk information (or, shallow parsing features) provides useful evidences about the boundaries of biomedical NEs. In the current work, we use chunk information of the current and/or the surrounding token(s).
7. **Dynamic feature**: Dynamic feature denotes the output tags *t*_*i*-3_*t*_*i*-2_*t*_*i*-1_, *t*_*i*-2_*t*_*i*-1_, *t*_*i*-1_of the word *w*_*i*-3_*w*_*i*-2_*w*_*i*-1_, *w*_*i*-2_*w*_*i*-1_, *w*_*i*-1_preceding *w*_*i*_in the sequence .
8. **Unknown token feature**: This is a binary valued feature that checks whether the current token was seen or not in the training corpus. In the training phase, this feature is set randomly.
9. **Word normalization**: We define two different types of features for word normalization. The first type of feature attempts to reduce a word to its stem or root form. This helps to handle the words containing plural forms, verb inflections, hyphen, and alphanumeric letters. The second type of feature indicates how a target word is orthographically constructed. Word shapes refer to the mapping of each word to their equivalence classes. Here each capitalized character of the word is replaced by 'A’, small characters are replaced by 'a’ and all consecutive digits are replaced by '0’. For example, 'IL’ is normalized to 'AA’, 'IL-2’ is normalized to 'AA-0’ and 'IL-88’ is also normalized to 'AA-0’.
10. **Head nouns**: Head noun is the major noun or noun phrase of a NE that describes its function or the property. For example, *transcription factor* is the head noun for the NE *NF-kappa B transcription factor*. In comparison to other words in NE, head nouns are more important as these play key role for correct classification of the NE class. In this work, we use only the unigram and bigram head nouns like *receptor*, *protein*, *binding protein* etc. For domain independence, we extract these head nouns only from the training data. A feature is defined that fires iff the current word or the sequence of words appears in either of these lists.
11. **Verb trigger**: These are the special types of verbs (e.g., *binds*, *participates* etc.) those occur preceding to NEs and provide useful information about the NE class. However, in the spirit of maintaining the domain independence of the system, we do not use a predefined list of trigger words. Based on their frequencies of occurrences, these trigger words are extracted automatically from the training corpus. A feature is then defined that fires iff the current word appears in the list of trigger words.
12. **Word class feature**: Certain kind of NEs, which belong to the same class, are similar to each other. The word class feature is defined as follows: For a given token, capital letters, small letters, numbers and non-English characters are converted to “A”, “a”, “O” and “-”, respectively. Thereafter, the consecutive same characters are squeezed into one character. This feature will group similar names into the same NE class.
13. **Informative words**: In general, biomedical NEs are too long and they contain many common words that are actually not NEs.
  For example, the function words such as *of*, *and* etc.; nominals such as *active*, *normal* etc. appear in the training data often more frequently but these don’t help to recognize NEs. In order to select the most important effective words, we first list all the words which occur inside the multiword NEs. Thereafter digits, numbers and various symbols are removed from this list. For each word (*w*_*i*_) of this list, a weight is assigned that measures how better the word is to identify and/or classify the NEs. This weight is denoted by *NEweight* (*w*_*i*_), and calculated as follows:1
  The effective words are finally selected based on the two parameters, namely *NEweight* and *number of occurrences*. The threshold values of these two parameters are selected based on some experiments. The words which have less than two occurrences inside the NEs are not considered as informative. The remaining words are divided into five classes. We compile five different lists for the above five classes of informative words. A binary feature vector of length five is defined for each word. If the current word in training (or, test) is found in any particular list then the value of the corresponding feature is set to 1. This feature is a modification to the one used in (Saha et al. 2009).
14. **Content words in surrounding contexts**: This is based on the content words in the surrounding context. We consider all unigrams in contexts  of *w*_*i*_(crossing sentence boundaries) for the entire training data. We convert tokens to lower case, remove stopwords, numbers, punctuation and special symbols. We define a feature vector of length 10 using the 10 most frequent content words. Given a classification instance, the feature corresponding to token *t* is set to 1 if and only if the context  of *w*_*i*_contains *t*. Evaluation results show that this feature is very effective to improve the performance by a great margin.
15. **Orthographic features**: We define a number of orthographic features depending upon the contents of the wordforms. Several binary features are defined which use capitalization and digit information. These features are: initial capital, all capital, capital in inner, initial capital then mix, only digit, digit with special character, initial digit then alphabetic, digit in inner. The presence of some special characters like (',’,'-’,'.’,')’,'(’ etc.) is very much helpful to detect NEs, especially in biomedical domain. For example, many biomedical NEs have '-’ (hyphen) in their construction. Some of these special characters are also important to detect boundaries of NEs. We also use the features that check the presence of ATGC sequence and stop words. The complete list of orthographic features is shown in Table 6.

**Table 6:** **Orthographic features**

Feature	Example	Feature	Example
InitCap	Src	AllCaps	EBNA, LMP
InCap	mAb	CapMixAlpha	NFkappaB, EpoR
DigitOnly	1, 123	DigitSpecial	12-3
DigitAlpha	2× NFkappaB, 2A	AlphaDigitAlpha	IL23R, EIA
Hyphen	-	CapLowAlpha	Src, Ras,Epo
CapsAndDigits	32Dc13	RomanNumeral	I, II
StopWord	at, in	ATGCSeq	CCGCCC, ATAGAT
AlphaDigit	p50, p65	DigitCommaDigit	1,28
GreekLetter	alpha, beta	LowMixAlpha	mRNA, mAb

We have used the C ^++^ based CRF ^++^ package^16^, a simple, customizable, and open source implementation of CRF for segmenting or labeling sequential data.

## Endnotes

^1^ftp://ftp.cs.utexas.edu/pub/mooney/bio-data/interactions.tar.gz

^2^ftp://ftp.ncbi.nlm.nih.gov/pub/tanabe/GENEATG.tar.gz

^3^ftp://ftp.cs.utexas.edu/pub/mooney/bio-data/interactions.tar.gz

^4^ftp://ftp.ncbi.nlm.nih.gov/pub/tanabe/GENEATG.tar.gz

^5^http://www-tsujii.is.s.u-tokyo.ac.jp/GENIA/ERtask/report.html

^6^http://www.nactem.ac.uk/tsujii/GENIA/ERtask/report.html

^7^ We use GENIA and JNLPBA to refer to the same corpus throughout the paper

^8^ftp://ftp.ncbi.nlm.nih.gov/pub/tanabe/GENEATG.tar.gz

^9^http://www.biocreative.org/news/biocreative-ii/

^10^http://crfpp.sourceforge.net

^11^http://chasen.org/~taku/software/yamcha/

^12^http://chasen.org/~taku/software/yamcha/

^13^http://chasen.org/~taku/software/TinySVM/

^14^ We also treat the beginning and internals (denoted by BIO labeling scheme) of a multiword NE as the separate classes

^15^http://www-tsujii.is.s.u-tokyo.ac.jp/GENIA/tagger

^16^http://crfpp.sourceforge.net
